# Characterization of an iron-inducible *Haemaphysalis longicornis* tick-derived promoter in an *Ixodes scapularis-*derived tick cell line (ISE6)

**DOI:** 10.1186/s13071-019-3574-9

**Published:** 2019-06-25

**Authors:** Emmanuel Pacia Hernandez, Kodai Kusakisako, Takeshi Hatta, Tetsuya Tanaka

**Affiliations:** 1 Laboratory of Infectious Diseases, Joint Faculty of Veterinary Medicine, Kagoshima University, 1-21-24 Korimoto, Kagoshima, 890-0056 Japan; 2 Department of Pathological and Preventive Veterinary Science, The United Graduate School of Veterinary Science, Yamaguchi University, Yoshida, Yamaguchi 753-8515 Japan; 3 Laboratory of Parasitology, Graduate School of Veterinary Medicine, Hokkaido University, Sapporo, Hokkaido 060-0818 Japan; 4 Department of Parasitology, Kitasato University School of Medicine, Kitasato, Minami, Sagamihara, Kanagawa 252-0374 Japan

**Keywords:** Tick, Promoter, Ferritin, Dual-reporter plasmid, Transfection, Post-transcriptional regulation, ISE6 cells

## Abstract

**Background:**

Ticks are important vectors of disease-causing pathogens. With the rise of resistance to chemical acaricides, alternative methods in tick control are warranted. Gene manipulation has been successful in controlling mosquitoes and mosquito-borne diseases and is now looked upon as a candidate method to control ticks and tick-borne pathogens. Our previous study has identified the *actin* and *ferritin* promoter regions in the *Haemaphysalis longicornis* tick.

**Results:**

Here, the *ferritin*-derived promoter from the *H. longicornis* tick was characterized *in silico*, and the core promoter sequences and some of its important components were identified. Several truncations of the promoter region were created and inserted to a reporter plasmid to determine the important components for its activity. The activities of the truncated promoters on the *Ixodes scapularis* tick cell line (ISE6) were measured *via* a dual luciferase assay using experimental and control reporter genes. To induce the promoter’s activity, transfected ISE6 cells were exposed to ferrous sulfate. The 639 nucleotides truncated promoter showed the highest activity on ISE6 cells when exposed to 1 mM ferrous sulfate.

**Conclusion:**

In this study, we characterized an iron-inducible tick promoter that could be a valuable tool in the development of a gene-manipulation system to control ticks and tick-borne pathogens.

**Electronic supplementary material:**

The online version of this article (10.1186/s13071-019-3574-9) contains supplementary material, which is available to authorized users.

## Background

Next to mosquitoes, ticks are considered to be the most important vectors of pathogens that cause human diseases [1]. At present, the main method of controlling tick infestation has been the use of chemical acaricides. Due to numerous reports of acaricide resistance in ticks, alternative approaches are warranted [2]. Gene-manipulation techniques have been used in mosquitoes and have resulted in a decrease in mosquito populations and in the transmission of mosquito-borne pathogens [3]. However, gene-manipulation techniques in ticks mainly involved RNA interference (RNAi). RNAi techniques for specific tick molecules have been used mostly in the analysis of tick biological function and the interaction of ticks and tick-borne pathogens but not as the main method to control them [4]. This may be because RNAi is low throughput, labor-intensive, and relatively slow to yield results [5]. Therefore, an alternative gene-manipulation technique such as introduction of foreign genes in the ticks, could be explored to control ticks and tick-borne pathogens.

*Haemaphysalis longicornis* is a hard tick that can be found in East Asia and Australia and that has recently attracted much attention because of reports of infestation with this species in several states in the USA [6]. It is also considered a vector of the severe fever with thrombocytopenia syndrome virus (SFTSV) affecting humans [7, 8]. Numerous studies on *H. longicornis*, from its survival against acaricides to its vector competence against several viruses, have already been conducted [9, 10]. It is necessary to control this tick species due to its effects on human and animal health and the economy. One possible method of tick and tick-borne disease control is the development of transgenic ticks.

The development of transgenic ticks requires an effective promoter derived from the organisms themselves. In mosquitoes, it is believed that the best candidate promoters for the expression of foreign genes are those that are inducible during blood-feeding due to their strength, tissue specificity, and synchrony of expression with the pathogen infection [11]. This could also be the case in ticks. Our previous study has identified two promoters from *H. longicornis*: the *actin* and *ferritin1* promoters. The *actin* promoter could effectively express the foreign gene in an *Ixodes scapularis* embryo-derived cell line (ISE6), but the ability of the *ferritin1* promoter to express a foreign gene was not demonstrated. It was hypothesized that this is due to the presence of iron-regulatory proteins (IRPs) in the untranslated region that prevents effective translation of the foreign gene [12]. On the other hand, our recent study showed a dose-dependent ferritin expression on a tick cell line after the addition of iron [13].

During blood-feeding, the actin gene and protein of ticks are constitutively expressed [14, 15]. In the same manner, the *ferritin1* mRNA is constitutively expressed, however, its protein is inducible by blood-feeding due to the presence of iron in the blood diet [14]. Therefore, the ferritin promoter could be a good candidate in the development of an inducible promoter system for the tick cell line that could be similar to the blood-feeding in ticks. Here, the promoter region of the *ferritin1* gene of *H. longicornis* (*HlFer1*) was analyzed and its activity demonstrated using the ISE6 cell line.

## Methods

### *In silico* analysis of *H. longicornis ferritin1* (*HlFer1*) promoter sequence

Analysis of the *HlFer1* promoter region was conducted to determine the functional region of the previously identified *HlFer1* promoter. Transcription starting site (TSS) analysis was done by first aligning the ESTs for *HlFer1* using MAFFT software (https://mafft.cbrc.jp/alignment/software/). Then a WebLogo image of the TSS was made (https://weblogo.berkeley.edu/logo.cgi). Using the neural network promoter program (http://www.fruitfly.org/seq_tools/promoter.html), the possible promoter regions were identified. Further characterization of the promoter regions was done using gene promoter-mining software (http://gpminer.mbc.nctu.edu.tw/).

### *Renilla* luciferase reporter construct

To establish a purely tick promoter reporter plasmid, the *H. longicornis actin* (*HlActin*) promoter was used to replace the SV40 promoter of the *Renilla luciferase* (hrLuc) of the pmirGLO plasmid vector (Promega, Madison, WI, USA). First, the previously identified *HlActin* promoter region was amplified from the previously constructed pmirGLO-*HlActin* pro [12] using a primer pair of HlActin-Renilla F and HlActin-Renilla R (Additional file 1: Table S1) by PCR with KOD-Plus-Neo (Toyobo, Osaka, Japan). After amplification, the PCR products were electrophorosed in 1.0% agarose gel. The PCR product was purified using a QIAquick Gel Extraction Kit (Qiagen, Hilden, Germany). The pmirGLO vector was then double-digested using *Kpn*I and *Pfm*I to remove the SV40 promoter. After double digestion, the plasmid vector was electrophoresed in 1.0% agarose gel and purified using a QIAquick Gel Extraction Kit (Qiagen). The digested vector and purified product were mixed with an In-Fusion HD cloning kit (Takara, Shiga, Japan). After the ligation of pmirGLO/*HlActin*-hrLuc, the plasmid was transformed into the *Escherichia coli* Stellar strain and purified using a Plasmid Midi Kit (Qiagen).

### Firefly *luciferase* construct

**Fig. 1:**
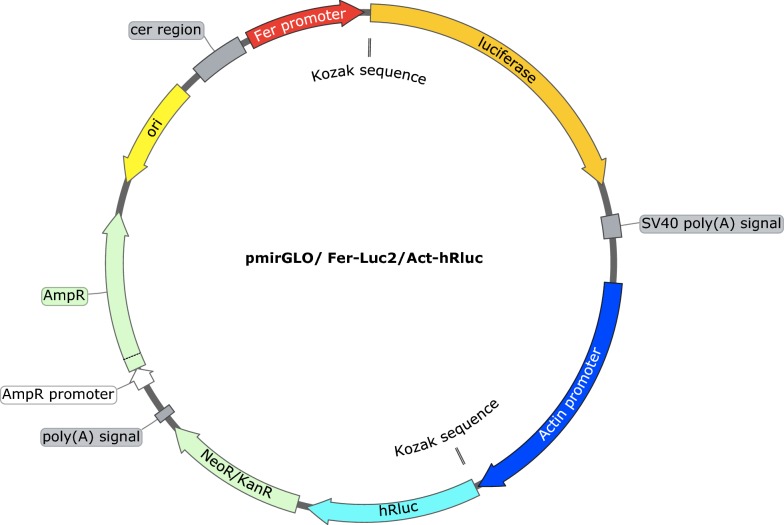
Schematic diagram of the constructed pmirGLO/HlFer Luc2/HlActin-hrLuc plasmid, created using a SnapGene Viewer. Luciferase (Luc2) indicates the firefly luciferase gene, while hrLuc is the *Renilla* luciferase gene. Ori indicates the origin of replication. The cer region is the stability region of the plasmid [37]. The Kozak sequences are consensus sequences that play a role in the translation process [38]. The poly (A) signal is for the termination of the transcription [39]

The previously constructed pmirGLO/*HlActin*-hrLuc was digested using *Bgl*II and *Hind*III at 37 °C for 2 h to remove the PGK promoter. After double digestion, the plasmid vector was electrophoresed in 1.0% agarose gel and purified using a QIAquick Gel Extraction Kit (Qiagen). The *HlFer1* promoter sequence was amplified from the previously constructed pmirGLO-*HlFer1* plasmid using KOD-Plus-Neo (Toyobo) with the indicated primer sets (Additional file 1: Table S1). The *HlFer1* promoter sequences were purified using a QIAquick Gel Extraction Kit (Qiagen). The purified vector was mixed with and ligated using an In-Fusion HD cloning kit (Takara). After ligation, the plasmids were transformed into the *E. coli* Stellar strain and purified using a Plasmid Midi Kit (Qiagen). For the pmirGLO-no promoter-Luc2/*HlActin*-hrLuc, the purified vector was allowed to self-ligate using DNA Ligation Kit Ver 2.1 (Takara) and incubated at 16 °C overnight. It was then transformed into the *E. coli* DH5α strain and purified using a Plasmid Midi Kit (Qiagen). Finally, the pmirGLO/*HlFer1*(F0-F4 and no promoter)-Luc2/HlAct-hrLuc plasmids were obtained. Plasmid inserts from the clones were sequenced from the beginning to the end of the target promoter sequence using a BigDye Terminator v. 3.1 Cycle Sequencing Kit (Applied Biosystems, Foster City, CA, USA) with sequencing primers derived from the pmirGLO vector. A diagram for the constructed plasmid was created using SnapGene Viewer software (http://www.snapgene.com) (Fig. 1).

### Tick cell culture and transient transfection of plasmid vectors

The ISE6 cell line from the embryo of *I. scapularis* was grown at 34 °C in L-15B medium (pH 6.4–6.6) with 10% fetal bovine serum, 5% tryptose phosphate broth, and 50 units/ml antibiotics-penicillin G and 50 μg/ml streptomycin [16, 17].

ISE6 cells were seeded in a 24-well plate at 0.5 ml/well of 1.0 ×  10^6^ cells/ml and incubated at 34 °C overnight. The plasmid vector (1.5 μg/well), 50 μl of Opti-MEM (Gibco, Grand Island, NY, USA), and 1.5 μl of Lipofectamine 2000 (Invitrogen, Carlsbad, CA, USA) were mixed and incubated at room temperature for 5 min. Then the incubated mixture was added to the medium in each well, and the cells were incubated at 34 °C for 2 days and replaced with different concentrations (0, 0.1, 1 and 2 mM) ferrous sulfate-enriched iron media, then further incubated for 4 days according to previous studies of ferritin induction [13]. The transfected cells were harvested and assayed for firefly and *Renilla* luciferase activities as described below.

### Dual luciferase assays

The firefly and *Renilla* luciferase activities were assayed using a Dual-Glo Luciferase Assay System with Passive Lysis Buffer (Promega) and the microplate reader (SH-9000Lab, Corona Electric, Ibaraki, Japan), following the manufacturer’s protocol. The firefly luciferase activity corresponding to the *Renilla* luciferase activity was calculated (relative luciferase activity).

### Construction of yellow fluorescent protein (Venus) expression plasmid vector using *HlFer1* promoter regions

Yellow fluorescent protein (Venus) expression vectors with an *HlFer1*(F2) promoter region were constructed. To produce a pmirGLO/*Fer1* (F2)-Venus plasmid, the *Fer1* (F2) promoter was amplified by PCR using KOD-Plus-Neo (Toyobo) with pmirGLO-HlFer2-Venus F and pmirGLO-HlFer2*-*Venus R primers, while the *Venus* gene was also amplified using pmirGLO-Venus F and pmirGLO Venus-*Xho*I R. These PCR products were purified using a QIAquick Gel Extraction Kit (Qiagen). The pmirGLO plasmid was double digested with *Bgl*II and *Xho*I to remove the PGK promoter and *luciferase* gene from the vector. The obtained vector and two purified products were mixed with an In-Fusion HD Cloning Kit (Takara) and incubated. After ligation, the plasmid was transformed into the *E. coli* Stellar cells and purified using a Plasmid Midi Kit (Qiagen). The pmirGLO/no promoter-Venus was constructed by blunt ligation of the purified *Venus* gene with the digested vector using DNA Ligation Kit Vector 2.1 (Takara).

### Transfection of Venus expression plasmid into ISE6 cells and comparison of promoter activities using fluorescence microscopy and western blotting

Transfection was performed as mentioned above. As a negative control, Lipofectamine 2000 (Invitrogen) was used without the plasmid. The previously synthesized pmirGLO-*HlActin*-Venus was used as a positive control. The incubated mixture was added to each well, and after 2 days, the media were replaced with 1 mM ferrous sulfate-enriched media and incubated at 34 °C for 4 days according to previous studies for promoter activity [5]. The transfected cells were observed under fluorescence microscopy (IX71, Olympus, Tokyo, Japan).

Another set of transfected cells was collected and subjected to western blotting as described by Kusakisako et al. [12]. Briefly, transfected cells were collected and suspended in phosphate-buffered saline (PBS) and sonicated at 45 kHz for 6 min using a VS-100III ultrasonic cleaner (AS ONE, Osaka, Japan), then centrifuged at 20,100×*g*. The supernatant was resolved in a 12% SDS-polyacrylamide gel electrophoresis (SDS-PAGE) under a reducing condition. The proteins were then transferred onto a polyvinylidene difluoride membrane (Immobilon-P; Millipore, Danvers, MA, USA). The membranes were then blocked at room temperature for 1 h with 0.3% skimmed milk in PBS containing 0.05% Tween 20 (PBS-T, blocking solution). After blocking, the membranes were incubated in a 1:1000 dilution anti-green fluorescent protein (GFP) pAb (MBL, Nagano, Japan) in a blocking solution at 4 °C overnight. The antiserum against recombinant *H. longicornis* β-tubulin [18] was used as a loading control. After incubation, the membranes were washed three times with PBS-T and then incubated with a 1:50,000 dilution of horseradish peroxidase (HRP)-conjugated goat anti-mouse or anti-rabbit IgG (Dako, Glostrup, Denmark) in a blocking solution at room temperature for 1 h. The membranes were then washed five times using PBS-T. After washing, the bands were detected using Amersham™ ECL™ Prime Western Blotting Detection Reagent (GE Healthcare, Buckinghamshire, UK) and viewed using FluorChem®FC2 software (Alpha Innotech, San Leandro, CA, USA).

### Statistical analysis

Statistical analyses were performed using STATA15.0 software. The data were initially checked for normality and homogeneity assumptions using the Shapiro-Wilk W-test for normality and Breusch-Pagan/Cook-Weisberg test for heteroskedasticity. A one-way analysis of variance (ANOVA) with Bonferroni multiple comparison *post-hoc* tests was applied. Statistical significance was set as *P < *0.05. Data presented are the results of at least two independent experiments.

## Results

### Analysis of the *Haemaphysalis longicornis ferritin1* promoter region sequence

**Fig. 2:**
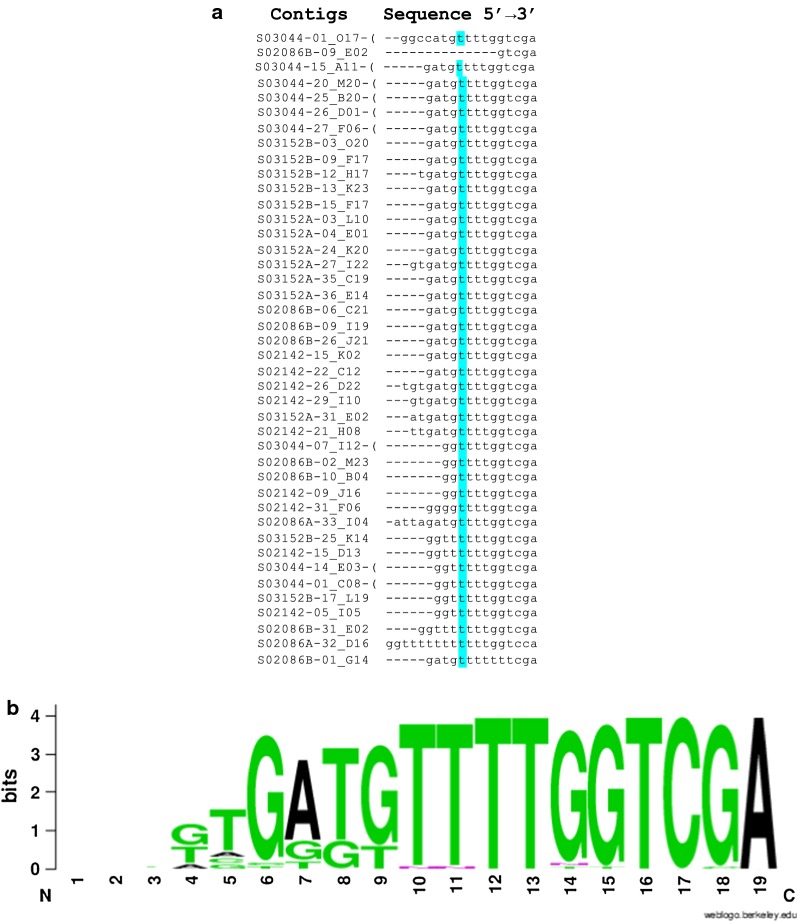
Prediction of the TSS of *HlFer1*. **a** Multiple-sequence alignment of the EST sequence of *HlFer1*. For easier alignment, sequences from the iron-responsive element (IRE) and downstream sequence were not included. Highlighted in blue is the predicted TSS. **b** WebLogo image of the TSS. The initial [G] of the 5′ termini were assumed to be the cap of the mRNA

**Fig. 3:**
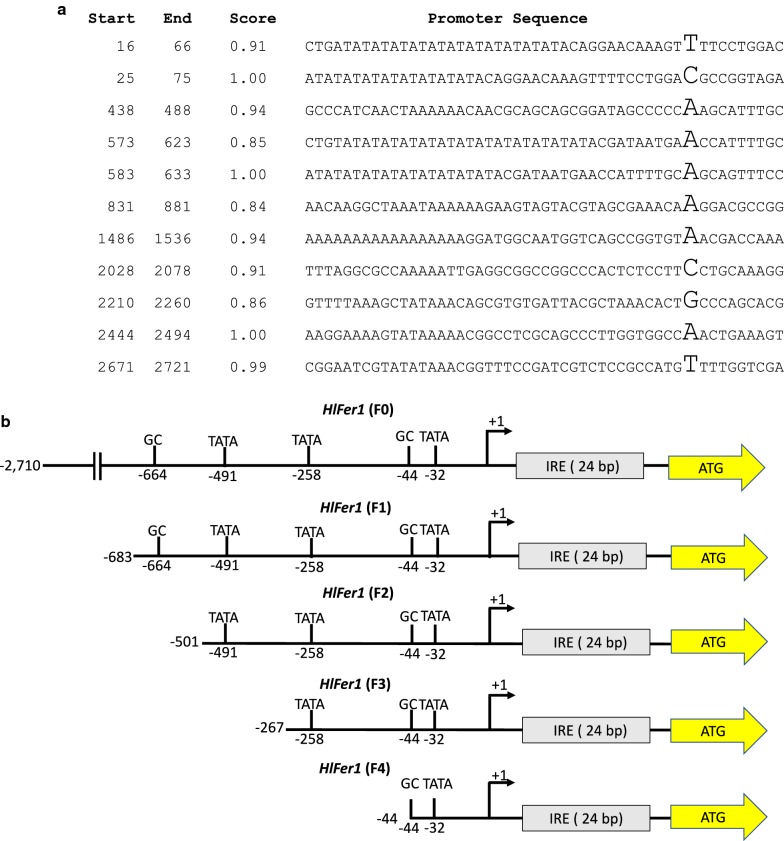
**a** Analysis of the predicted *HlFer1* promoter sequences using the neural network promoter program. Beginning and ending nucleotide sequence of the predicted promoter are indicated from the whole promoter sequence. The score indicates the likelihood of the sequence to be the promoter: values closer to 1 indicate higher likelihood. The predicted TSSs in each predicted promoter sequences are shown in a larger font. **b** Graphical representation of the truncated *HlFer1* promoter sequences. Predicted TATA and GC boxes are indicated. Positions are relative to the transcription starting site (TSS)

To determine the core promoter region of the previously identified *HlFer1* promoter sequence [12], analysis of the promoter sequence was performed. Initially, the transcription starting site (TSS) was identified. Alignment of *HlFer1* candidate sequences from the expressed sequence tag (EST) library was performed using MAFFT alignment software (Fig. 2a). Based on the alignment, a WebLogo image of the TSS region was made (Fig. 2b). To further investigate the core promoter sequence containing the TSS region of the *HlFer1* gene, the sequence of the *HlFer1* promoter region was subjected to a neural network for promoter prediction (NNPP) program in the Berkeley Drosophila Genome Project. Based on the alignment and the prediction software, the [T] nucleotide was the estimated TSS among the sequences (Figs. 2 and 3a).

The NNPP program also predicted several promoter sequences from the *HlFer1* promoter region (Fig. 3a). This promoter region was also evaluated using promoter-mining software to determine the important components of the promoter regions, such as the TATA and GC box, and match them with the predicted promoter sequence. Based on the findings, five promoter sequences were identified as candidates for an optimal promoter region of *HlFer1* and tentatively labeled as *HlFer1* (F0) for the 2848 nucleotide (nt) whole promoter, *HlFer1* (F1) for the 821 nt promoter, *HlFer1* (F2) for the 638 nt promoter, *HlFer1* (F3) for the 450 nt promoter, and *HlFer1* (F4) for the 185 nt promoter (Fig. 3b). These results indicate that the identified promoter region has a high possibility of being the true promoter region of *HlFer1.*

### Evaluation of the *HlFer1* promoter sequences activity in ISE6 cells using a dual luciferase assay

**Fig. 4:**
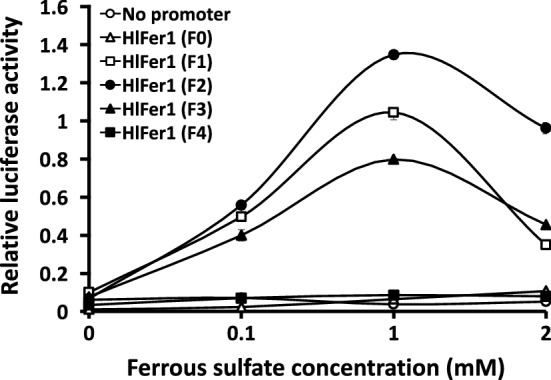
Evaluation of the *HlFer1* promoter truncates using relative luciferase activity in ISE6 cells enriched with different concentrations of ferrous sulfate. The pmirGLO plasmids containing *HlFer1* promoter truncates were transfected to ISE6 cells using a 1:1 Lipofectamine 2000 transfection reagent. The cells were incubated for 2 days, replaced with different concentrations of ferrous sulfate (0, 0.1, 1 and 2 mM) enriched media, incubated for another 4 days, and then tested for luciferase activity using a Dual-Glo Luciferase Assay System following the manufacturer’s protocol

Promoter-replacing expression vectors were constructed based on a pmirGLO plasmid vector with the phosphoglycerate kinase (PGK) promoter as the original promoter. The PGK promoter was replaced by truncated *HlFer1* promoters to evaluate the promoter activity of the candidate *HlFer1* promoter sequences in ISE6 cells. The previously identified *HlActin* promoter was used instead of the SV40 promoter to maintain consistency in using a tick-derived promoter. It has been previously demonstrated that the interaction between the iron-responsive element (IRE) and IRP may have resulted in the depressed activity of the *HlFer1* promoter. Therefore, different concentrations of ferrous sulfate were used in the media to liberate the IRP from the IRE for translation induction. A dose-dependent luciferase activity for the different truncated promoters up to 1 mM ferrous sulfate was observed. With 2 mM ferrous sulfate, decreased activity was observed at the *HlFer1* (F1), *HlFer1* (F2), and *HlFer1* (F3) truncates (Fig. 4, Additional file 1: Table S2). Among the promoters, the *HlFer1* (F2) possessed the highest luciferase activity in all of the ferrous iron-enriched media, followed by the *HlFer1* (F1) and *HlFer1* (F3) truncates, except at a 2 mM ferrous sulfate concentration, wherein *HlFer1* (F3) has a slightly higher activity than *HlFer1* (F1). The *HlFer1* (F0) and *HlFer1* (F4) sequences exhibited the lowest relative luciferase activity, and their relative luciferase activity did not differ significantly (Fig. 4, Additional file 1: Table S2). These results demonstrate that the *HlFer1* (F4) being the smallest truncated promoter and still exhibited activity could be the core promoter region, while the *HlFer1* (F2) showed optimal promoter activity in the presence of 1 mM ferrous sulfate (*F*_(15,24)_ = 540.83, *P < *0.0001).

### Demonstration of the promoter activity of the *HlFer1* promoter regions in ISE6 cells under fluorescence microscopy and western blotting

**Fig. 5:**
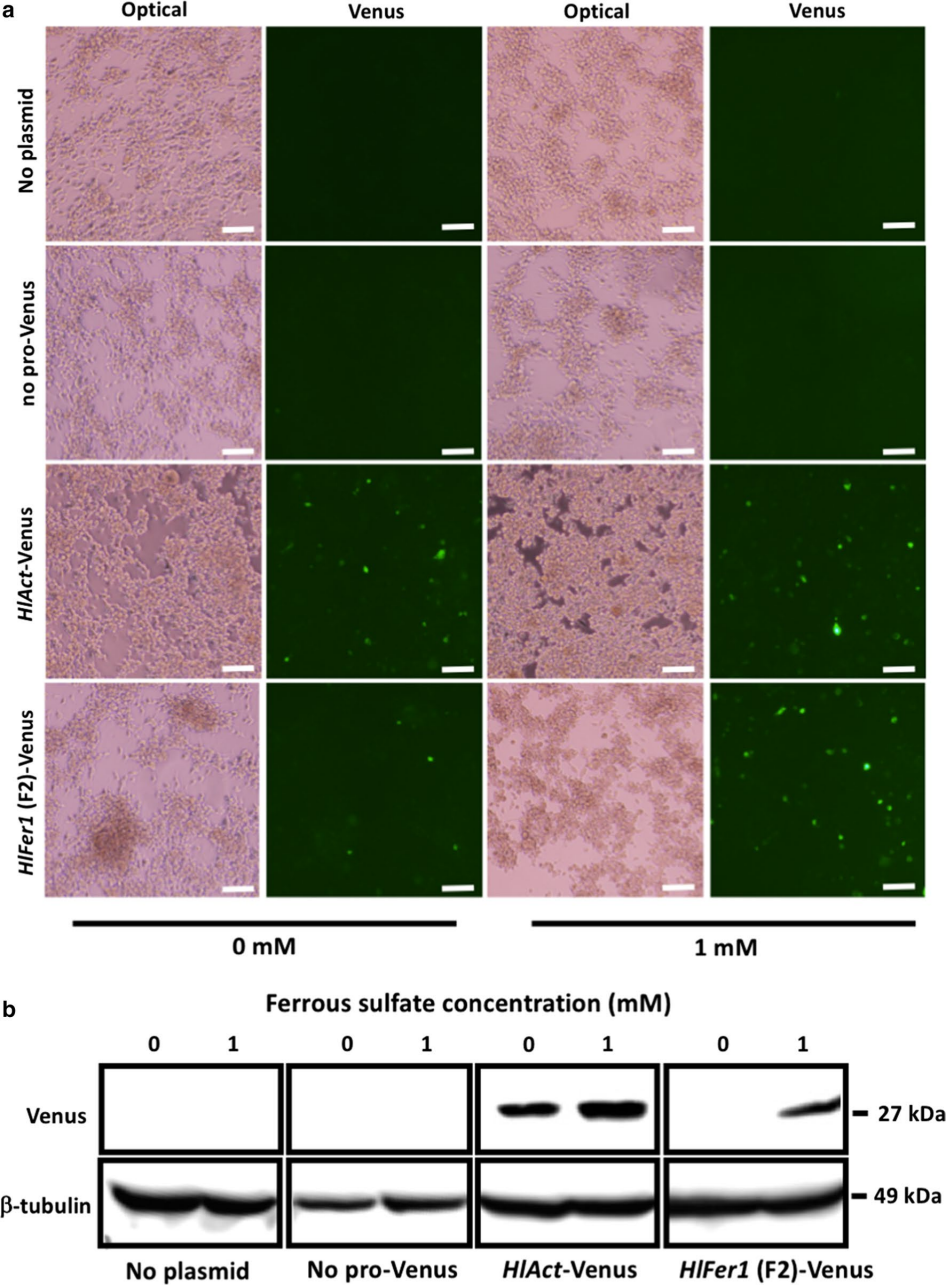
Observation of *HlFer1* (F2) promoter activity using Venus in ISE6 cells *via* fluorescence microscopy (**a**) and western blotting (**b**). The pmirGLO-no promoter-Venus, pmirGLO-*HlAct* pro-Venus, and pmirGLO-*HlFer1*(F2)-Venus plasmids (1.5 μg) with 1.5 μl Lipofectamine 2000 were transfected into 1 × 10^6^ ISE6 cells in a 24-well plate. Media enriched with 1 mM ferrous sulfate were used 2 days after transfection. Cells were observed under a fluorescence microscope after 4 days of medium enrichment. Western blotting results are representative data of three separate experiments. *Scale-bars*: 100 μm

Expression vectors of a yellow fluorescent protein, Venus, were constructed to evaluate the *HlFer1* (F2) promoter activity under fluorescence microscopy to show the activity of this truncated promoter. The previously constructed pmirGLO-*HlAct* pro-Venus and pmirGLO-*HlFer1*(F2)-Venus, wherein the PGK promoter and *luciferase* gene were replaced by the *HlFer1*(F2) promoter and the *Venus* gene, respectively, in the pmirGLO plasmid were evaluated. The mentioned vectors were transfected to ISE6 cells, and then the cells were enriched with 1 mM ferrous sulfate. The fluorescence of Venus was observed under fluorescence microscopy at day 4 after ferrous sulfate enrichment. The fluorescence microscopy test showed that the addition of 1 mM ferrous sulfate resulted in the increased fluorescence intensity of pmirGLO-*HlFer1*(F2)-Venus transfected cells; however, the addition of 1 mM ferrous sulfate did not affect the intensity of pmirGLO-*HlAct* pro-Venus transfected cells. No apparent difference in intensity was observed between pmirGLO-*HlAc*t pro-Venus and pmirGLO-*HlFer1*(F2)-Venus transfected ISE6 cells (Fig. 5a). Venus proteins were also investigated using western blotting. Western blot analysis showed that the addition of 1 mM ferrous sulfate to the ISE6 cells transfected with pmirGLO-*HlFer1*(F2)-Venus resulted in expression of the Venus protein in ISE6 cells (Fig. 5b). These results demonstrate that the promoter activity for a foreign gene is not limited to the *luciferase* gene but also occurs in other genes, such as the *Venus* gene.

## Discussion

Genetic manipulation of mosquitoes, particularly the introduction of foreign gene, has been shown to impair the transmission of malaria parasites [11, 19]. The development of ticks that possess genes that could affect the infection and transmission of tick-borne pathogens, then, could be explored as a promising strategy to control tick-borne diseases. Since gene regulation is a critical, well-coordinated, and complex process, an effective promoter is essential for the expression of a foreign gene into the target organism [12]. An inducible promoter is also important in the development of a tunable recombinant expression system [20, 21]. Recombinant proteins are important in the development of tick control strategies especially in vaccine design [22]. Insect cell lines are frequently utilized in recombinant protein production [23]. This strategy could be explored in tick cell lines in the development of expression system for tick-specific recombinant proteins.

Promoters are considered to be gene-regulatory sequences found immediately upstream of the TSS [24]. In this study, the promoter region of *HlFer1* was characterized for its possible use in the development of transgenic ticks and recombinant protein expression system in tick cell lines.

The core promoter is the minimal DNA sequence that is sufficient to initiate gene transcription [25]. It is where the transcription machinery is assembled [26]. The core promoter is generally 50 to 100 nt within the TSS [24]. Using the EST database promoter analysis software, the TSS [T] is predicted to be 138-bp upstream of the open reading frame sequence in the *HlFer1* gene. The TSS is included in the initiator element, which is, in turn, a key feature of the core promoter sequence [25]. Aside from the initiator element, another common core promoter element is the TATA box [26]. In metazoans, the TATA box is usually located around 30 nt upstream of the TSS, wherein the optimal spacing between the +1 TSS and the first T is around 30–31 nt upstream [25, 26]. The TATA sequence serves as the binding site for the transcription factor II D (TFIID). In this study, the TATA box was predicted to be 32 nt upstream of the TSS (Fig. 3b). Based on these results, the F4 sequence promoter was hypothesized as part of the core promoter region of the *HlFer1* promoter.

*In vitro* studies have shown that the core promoter is sufficient to initiate promoter activity, but an upstream promoter sequence may be necessary to generate activity *in vivo* [27]. The upstream sequence is also known as the extended promoter sequence. The extended promoter sequence is considered to contain regulatory elements that can modulate the downstream gene sequences [24, 26]. Therefore, several *HlFer1* sequences upstream of the core promoter region were evaluated using a reporter assay to determine the promoter sequence with the optimal activity (Fig. 3b).

A dual reporter assay, such as the dual luciferase reporter assay, has been commonly used in cell lines to evaluate promoter activity [12, 28, 29]. The use of a dual reporter system has been very advantageous in measuring gene expression. It is sensitive, allows experimental variations due to the presence of a control reporter gene, and has a high-throughput platform [30]. The dual luciferase assay has also been shown to be of value in tick cell lines [5] and has been used in evaluation of tick promoter. In this experiment, we used the previously identified *HlAct* promoter sequence from the *H. longicornis* tick [12] as the promoter of the control reporter gene and the *HlFer1* promoter sequences for the experimental reporter gene. Since there are no available cell lines for the *H. longicornis* tick, the embryo-derived *I. scapularis* cell line (ISE6) was utilized to evaluate the core promoter sequence of the different candidates in the *HlFer1* promoter sequences since the activity of *HlAct* and *HlFer1* promoter has already been established in this tick cell line [12].

Although the *HlFer1* promoter was tested for activity in our previous study, it showed depressed or no transcriptional activity [12]. This could be attributed not to the absence of transcriptional activity but rather to the presence of an IRE in the *HlFer1* mRNA, which interacts with the IRP that hinders the protein translation. On a previous study, an increasing HlFer1 protein expression was observed during blood-feeding despite no difference in the expression of *HlFer1* mRNA expression. It was then hypothesized that the increasing expression was due to the liberation of IRP from IRE due to the increased iron in the tick blood meal [14]. The same mechanism of intracellular ferritin protein expression was also proposed in *Ixodes* ticks [31]. In our recent study on the ISE6 cell line, Fer1 protein expression was induced by the addition of 0.1 to 2 mM ferrous sulfate to the media and incubation for at least 2 days [13]. It could be safe to assume therefore that the mechanism of intracellular ferritin translation in *Haemaphysalis* and *Ixodes* ticks, as well as *Ixodes* cells are all regulated by iron concentration. Therefore, ferrous sulfate enrichment on *HlFer1* promoter transfected ISE6 cells was utilized to determine whether it would result in luciferase activity due to liberation of the IRP and eventually, protein translation. A dose-dependent increase in activity was observed to peak in 1 mM ferrous sulfate-enriched media. The lower luciferase activity with 2 mM ferrous sulfate could be attributed to the possible increased cell mortality at the increased ferrous concentration, as was observed in our previous study [13]. With 1 mM ferrous sulfate, the *HlFer1* (F2) promoter showed the highest activity, followed by *HlFer1* (F1), *HlFer1* (F3), *HlFer1* (F4), and *HlFer1* (F0), with *HlFer1* (F4) and *HlFer1* (F0) not differing significantly, thus strengthening our hypothesis that the *HlFer1* (F4) promoter is the core promoter region. Since we consider this region to be the core promoter region, its activity could also be considered the basal activity of the promoter region [24]. The increased promoter activity of the *HlFer1* (F3) and *HlFer1* (F2) promoter regions could indicate that these extended regions contain the enhancers or positive regulatory elements. The decrease in the activity of the *HlFer1* (F1) and especially the *HlFer1* (F0) region could indicate that these regions contain the silencers or negative regulatory elements. This is consistent with human cell lines, in which the − 350 to − 40 nt region showed enhanced transcriptional activity, while the region further upstream, especially -500 to -1000 nt, showed a decline in transcriptional activity [24]. This is also consistent with tick promoters, wherein the promoter region − 649 nt from the TSS of the ribosomal protein L4 (rpL4) promoter of *Rhipicephalus microplus* showed a 17-fold higher promoter activity when compared with the 1191 nt originally cloned promoter. Meanwhile, a three-fold increase was observed when the − 1005 to − 495 sequences were deleted from the *EF1-α* promoter of the same tick species, indicating the presence of repressive elements in the upstream sequences of the promoters [5].

To further confirm the activity of the gene-expression vectors in ISE6 cells, *Venus* gene-expression vectors (pmirGLO/*HlAct*-Venus and pmirGLO/*HlFer1*(F2)-Venus) were constructed. More intense Venus fluorescence within transfected cells of the ferrous sulfate-enriched ISE6 cells was detected in pmirGLO-*HlFer1* (F2)-Venus transfected groups as compared to those without ferrous sulfate enrichment. Meanwhile, no difference in the ferrous sulfate-enrichment and non-enrichment was observed on pmirGLO-*HlAct* pro-Venus transfected cells. It would then be of interest to know if this Fer1 promoter sequence could also be found in *I. scapularis* and would exhibit same or higher activity. Nonetheless, we believed that the *HlFer1* promoter sequence maintained its promoter activity. These results also demonstrate the iron inducibility of the activity of the ferritin derived promoter.

## Conclusions

Precise spatial and temporal gene-expression and gene-modification control is necessary in the study of several gene functions. An inducible system of gene expression has been a valuable tool in the conduct of such study [32]. The use of iron as an inducing agent for promoter activity has already been demonstrated in *Trichomonas vaginalis* [33, 34]. Ticks, being obligate hematophagous parasites, are exposed to large amounts of iron and have developed mechanisms to overcome iron-related toxicity [35, 36]. We took advantage of presence of an IRE in the intracellular ferritin mRNA to identify and develop an iron-inducible promoter that could be used in tick cell line. Since many tick-borne pathogens are acquired during blood-feeding, a blood-feeding inducible tick promoter is necessary to the development of transgenic ticks for tick-borne pathogen control. Since blood-feeding would be difficult to simulate in tick cells, an increased iron environment would be a good alternative, since there is also an increased iron intake during blood-feeding. In this case, an iron-inducible promoter could be a valuable tool in such studies. The promoter could then be used in cell lines for the study of antimicrobial peptides against tick-borne pathogens that could be expressed during blood-feeding in the development of transgenic ticks. Aside from the abovementioned, this promoter could be applied for the development of an expression system that could be used to produce recombinant proteins that are tick-specific.

## Additional file

**Additional file 1: Table S1.** Oligonucletiode primer sequences used for construction of the plasmids. **Table S2.** Relative luciferase activity of the different *HlFer1* promoter truncates in ISE6 cells exposed to different concentrations of ferrous sulfate.

## Data Availability

All data generated or analyzed during this study are included in this published article and its additional file.

## Abbreviations

ISE6: *Ixodes scapularis* embryo-derived tick cell line
RNAi: RNA interference
HlFer1: *Haemaphysalis longicornis* ferritin 1
TSS: transcription starting site
EST: expressed sequence tags
NNPP: neural network for promoter prediction
PGK: phosphoglycerate kinase
IRE: iron-responsive element
IRP: iron-regulatory protein
TFIID: transcription factor II D
PBS: phosphate-buffered saline
HRP: horseradish peroxidase
ANOVA: analysis of variance
Luc2: firefly luciferase
GFP: green fluorescent protein
