# Supplementary material for: Characterization of an iron-inducible Haemaphysalis longicornis tick-derived promoter in an Ixodes scapularis-derived tick cell line (ISE6)
Source: Parasit Vectors. 2019 Jun 25;12:321. doi: 10.1186/s13071-019-3574-9 (PMC6593522; doi:10.1186/s13071-019-3574-9)
Supplement: Supplementary file 1 — Additional file 1: Table S1. Oligonucletiode primer sequences used for construction of the plasmids. Table S2. Relative luciferase activity of the different HlFer1 promoter truncates in ISE6 cells exposed to different concentrations of ferrous sulfate. [file 13071_2019_3574_MOESM1_ESM.docx]

**Additional file 1**

**Table S1.** Oligonucletiode primer sequences used for construction of the plasmid

| Primer | Sequence (5’→3’) |
| --- | --- |
| pmirGLO-HlActin-Renilla F | AACTTGGTTAGGTACGGCTTCGGACGAAGGCCA |
| pmirGLO-HlActin-Renilla R | CCATGGTGGCTCCAGGTTGACTGTTTAGCTGCAC |
| pmirGLO-HlFer0-Luc2 F | GGCGTAGAGGATCGATCCCGGTTCGACACCCTG |
| pmirGLO-HlFer0-Luc2 R | CCGGATTGCCAAGCTTTTCGTCGGTTATTTCCGG |
| pmirGLO-HlFer1-Luc2 F | GGCGTAGAGGATCGATTTAGGCGCCAAAAATTGAG |
| pmirGLO-HlFer1-Luc2 R | The same reverse primer sequence of pmirGLO-HlFer0-Luc2R |
| pmirGLO-HlFer2-Luc2 F | GGCGTAGAGGATCGAGTTTTAAAGCTATAAACAGCG |
| pmirGLO-HlFer2-Luc2 R | The same reverse primer sequence of pmirGLO-HlFer0-Luc2R |
| pmirGLO-HlFer3-Luc2 F | GGCGTAGAGGATCGAAAGGAAAAGTATAAAAACGGC |
| pmirGLO-HlFer3-Luc2 R | The same reverse primer sequence of pmirGLO-HlFer0-Luc2R |
| pmirGLO-HlFer4-Luc2 F | GGCGTAGAGGATCGAGCGGCGGAATCGTATATAA |
| pmirGLO-HlFer4-Luc2 R | The same reverse primer sequence of pmirGLO-HlFer0-Luc2R |
| pmirGLO-HlFer0-Venus F | The same forward primer sequence of pmirGLO-HlFer0-Luc2F |
| pmirGLO-HlFer0-Venus R | GCCCTTGCTCACCATTTTCGTCGGTTATTTCCGG |
| pmirGLO-HlFer2-Venus F | The same forward primer sequence of pmirGLO-HlFer2-Luc2F |
| pmirGLO-HlFer2-Venus R | The same reverse primer sequence of pmirGLO-HlFer0-VenusR |
| pmirGLO-Venus F | ATGGTGAGCAAGGGCGAGGAG |
| pmirGLO-Venus-*Xho*I R | GACTCTAGACTCGAGTTACTTGTACAGCTCGTCC |
| pmirGLO-sequence F | GTACCCTCTGGTTGCATAGGT |
| pmirGLO-sequence R | AGCCCATAGCGCTTCATAGC |

Hl, *Haemaphysalis longicornis*; Fer, ferritin1; Luc2, firefly luciferase.

Underline denotes the restriction enzyme recognition site mentioned in the primer name.

**Table S2.** Relative luciferase activity of the different *HlFer1* promoter truncates in ISE6 cells exposed to different concentrations of ferrous sulfate

|  | **Promoter** | | | | | |
| --- | --- | --- | --- | --- | --- | --- |
| **Fe_2_SO_4_ concentration (mM)** | **No promoter** | **Fer-F0** | **Fer-F1** | **Fer-F2** | **Fer-F3** | **Fer-F4** |
| **0** | 0.06 ± 0.000^ab^ | 0.01 ± 0.000^c1^ | 0.10 ± 0.000^b^ | 0.07 ± 0.003^ab^ | 0.08 ± 0.003 ^ab^ | 0.03 ± 0.001^ac1^ |
| **0.1** | 0.07 ± 0.005^a^ | 0.02 ± 0.000^b12^ | 0.50 ± 0.039 | 0.56 ± 0.019 | 0.40 ± 0.030 | 0.07 ± 0.001^ab12^ |
| **1** | 0.04 ± 0.007^a^ | 0.06 ± 0.004^ab23^ | 1.04 ± 0.009 | 1.35 ± 0.002 | 0.80 ± 0.001 | 0.09 ± 0.000^b2^ |
| **2** | 0.05 ± 0.010^a^ | 0.11 ± 0.008^b3^ | 0.35 ± 0.022 | 0.96 ± 0.030 | 0.46 ± 0.005 | 0.08 ± 0.027 ^ab2^ |

Rows with the same letter superscript and columns with the same numerical subscript are not significantly different at *P*<0.05.
